# Conformation‐specific antibodies against multiple amyloid protofibril species from a single amyloid immunogen

**DOI:** 10.1111/jcmm.14119

**Published:** 2019-01-20

**Authors:** Alessandra Bonito‐Oliva, Sophia Schedin‐Weiss, Shahab S. Younesi, Ann Tiiman, Carolina Adura, Navid Paknejad, Matt Brendel, Yevgeniy Romin, Ronald J. Parchem, Caroline Graff, Vladana Vukojević, Lars O. Tjernberg, Lars Terenius, Bengt Winblad, Thomas P. Sakmar, W Vallen Graham

**Affiliations:** ^1^ Laboratory of Chemical Biology & Signal Transduction The Rockefeller University New York City New York; ^2^ Division of Neurogeriatrics, Department of Neurobiology, Care Sciences and Society Center for Alzheimer Research, Karolinska Institutet Solna Sweden; ^3^ Department of Neuroscience, Stem Cells and Regenerative Medicine Center Baylor College of Medicine Houston Texas; ^4^ Department of Clinical Neuroscience Center for Molecular Medicine, Karolinska Institutet Stockholm Sweden; ^5^ High Throughput and Spectroscopy Resource Center, The Rockefeller University New York City New York; ^6^ Molecular Cytology Core Facility Memorial Sloan‐Kettering Cancer Center New York City New York; ^7^ Theme Aging Karolinska University Hospital Huddinge Stockholm Sweden

**Keywords:** Alzheimer’s disease, amyloid, amyloid aggregation, chaperone‐like amyloid‐binding protein, conformation‐sensitive, monoclonal antibody, protofibril

## Abstract

We engineered and employed a chaperone‐like amyloid‐binding protein Nucleobindin 1 (NUCB1) to stabilize human islet amyloid polypeptide (hIAPP) protofibrils for use as immunogen in mice. We obtained multiple monoclonal antibody (mAb) clones that were reactive against hIAPP protofibrils. A secondary screen was carried out to identify clones that cross‐reacted with amyloid beta‐peptide (Aβ42) protofibrils, but not with Aβ40 monomers. These mAbs were further characterized in several in vitro assays, in immunohistological studies of a mouse model of Alzheimer's disease (AD) and in AD patient brain tissue. We show that mAbs obtained by immunizing mice with the NUCB1‐hIAPP complex cross‐react with Aβ42, specifically targeting protofibrils and inhibiting their further aggregation. In line with conformation‐specific binding, the mAbs appear to react with an intracellular antigen in diseased tissue, but not with amyloid plaques. We hypothesize that the mAbs we describe here recognize a secondary or quaternary structural epitope that is common to multiple amyloid protofibrils. In summary, we report a method to create mAbs that are conformation‐sensitive and sequence‐independent and can target more than one type of protofibril species.

## 1 INTRODUCTION

Aggregation of proteins or peptides into amyloid fibrils is a characteristic pathological feature observed in many different diseases including type 2 diabetes mellitus (T2DM), Alzheimer's disease (AD), Parkinson's disease (PD), and Huntington's disease (HD).^1^, ^2^, ^3^ Amyloid aggregates present in the brain are associated with a reduction in the efficiency of coordinated synaptic transmission, loss of synaptic plasticity and contribute to cognitive impairment^4^, ^5^ in AD^6^, ^7^, ^8^ and other so‐called tauopathies,^9^, ^10^ PD,^11^ HD^12^ and frontotemporal lobar degeneration (frontotemporal dementia, clinical amyotrophic lateral sclerosis and motor neuron disease).^13^, ^14^ Misfolded protein aggregates outside of the central nervous system result in amyloidosis syndromes, such as light‐chain amyloidosis, familial amyloid cardiomyopathy, familial amyloid polyneuropathy^15^ and T2DM.^16^

Amyloid proteins or peptides polymerize to form a cross‐β sheet structure and progressively self‐aggregate into soluble protofibrils, insoluble fibrils and eventually deposit as amyloid plaques in tissue.^17^ However, normally folded species of these proteins or peptides have important biological functions, the amyloid fibrils and their prefibrillar aggregates exhibit toxicity.^18^, ^19^ Antibodies that lead to clearance of the toxic forms of amyloid are likely more useful than those that target the monomeric amyloidogenic species. In fact, accumulating evidence suggests that prevention of aggregation of pathogenic amyloid species would prevent disease progression.^20^

Clinical applications of antibodies that target amyloid conformations are primarily limited to AD. Passive immunization with monoclonal antibodies (mAbs) directed at amyloid beta‐peptide (Aβ42) aggregates has shown interesting preliminary results.^21^ BAN2401 (BioArtic Neuroscience AB, Eisai Co., Ltd., currently in phase II) was obtained by immunizing mice with Aβ42 (E22G) protofibrils and recognizes early aggregates with low affinity for fibrils or monomers.^22^, ^23^ Crenezumab (Genentech, Inc, currently in phase III) binds the middle domain and shows similar binding for Aβ42 monomeric, oligomeric and fibrillar species.^24^ Finally, produced through a “reverse translational medicine” approach, aducanumab (Biogen, Inc, currently in phase III) was isolated from B‐cells of healthy advanced‐age donors, who are hypothesized to harbour naturally developed antibodies against Aβ. Aducanumab selectively targets aggregates and dose‐dependently reduces amyloid deposition.^25^, ^26^

We recently described a platform technology based on the use of the chaperone‐like amyloid‐binding protein (CLABP) NUCB1 to “cap,” detoxify, and stabilize soluble intermediate protofibrils originating from various amyloidogenic proteins, such as Aβ42, α‐synuclein, transthyretin and the human islet amyloid polypeptide (hIAPP).^27^ Based on the hypothesis that there are common core protofibril conformations we tested the possibility that our technology could be used to develop antibody tools to detect these similarities. Here, we demonstrate that NUCB1‐capped hIAPP protofibrils can be used as immunogen to produce mAbs against protofibrils derived from a different amyloid protein, Aβ42. We show that NUCB1‐capped amyloid could serve as a platform technology for the discovery of therapeutic antibodies that bind elements unique to structured amyloid intermediates.

## 2 MATERIALS AND METHODS

### 2.1 Peptide preparation

The hIAPP (Phoenix Pharmaceutics) was solubilized in HFIP at 1 µg/µl, dried and stored at −80°C. On the day of the experiment, hIAPP was solubilized in 20 mmol/L sodium phosphate buffer, pH 7.6 to a final concentration of 10 µmol/L, incubated at 25°C and tested at different time points. Aβ40 or Aβ42 (American Peptide) synthetic peptide was solubilized in HFIP at 1 µg/µl, dried and stored at −80°C. On the day of the experiment, Aβ42 was reconstituted in 2 mmol/L NaOH to 1 µg/µl, dried and diluted in 20 mmol/L sodium phosphate buffer, pH 8.0 to a final concentration of 10 µmol/L. The peptide was incubated at 37°C and tested at different time points. The Ca^2+^‐free *s*NUCB1 mutant (*mt*NUCB1) was engineered as previously described.^27^

### 2.2 Immunogen preparation and immunization strategy

The *mt*NUCB1‐capped hIAPP complex was prepared by co‐incubating *mt*NUCB1 (10 µmol/L) and hIAPP (33 µmol/L) peptide at 37°C for 3 hours while stirred. The capped‐protofibril containing solution was then applied to a Superdex200 26/60 PG size exclusion chromatography (SEC) column (GE Healthcare, Piscataway, NJ) equilibrated with buffer (20 mmol/L sodium phosphate, pH 7.6, 150 mmol/L NaCl). The main peak was collected, characterized^27^ and used as immunogen. ImmunoMax mice (n = 3) were immunized with consecutive boosts of NUCB1‐hIAPP complex and produced robust titer to the immunogen.

### 2.3 Dot blot

The recombinant hIAPP peptide was incubated at the monomeric concentration of 60 µmol/L for 0, 1, 5, 10, 20 or 30 minutes at room temperature (RT) and successively 50 µl of a 1:10 dilution of each time point was blotted onto an activated polyvinylidene fluoride membrane (1 µg per spot) allowing the samples to dry and then blocking with 5% BSA solution in TBS (20 mmol/L Tris–HCl, pH 7.5, 150 mmol/L NaCl) at RT. The membrane was incubated with our mAbs (4A8.E11, 4B1.H9, 3F2.E10, 5C9.A2, 1:100), the anti‐Amylin (hIAPP) antibody (Abcam, 1:1000) and the polyclonal anti‐oligomer A11 antibody (ThermoFisher Scientific 1:1000) diluted in TBS for 1 hour at RT followed by three washes with TBS‐T (TBS and 0.05% Tween‐20). The membranes were then incubated with horseradish‐peroxidase‐conjugated anti‐rabbit secondary antibody (1:10 000) in TBS for 30 minutes at RT followed by two washes with TBS‐T and a subsequent wash with TBS. The blots were developed using the SuperSignal West Femto Maximum Sensitivity Substrate (Thermo Scientific).

### 2.4 Enzyme‐linked immunosorbent assay

#### 2.4.1 Hybridoma supernatant screening

Hybridomas were created using the HTP Hybridoma production process at AbPro Labs (Lexington, MA). The supernatants were screened for their binding to the NUCB1‐hIAPP complex and NUCB1 alone through direct ELISA. Maxisorp 96‐well plates (NUNC) were coated for 2 hours at room temperature (RT) with 50 µl of either hIAPP‐NUCB1 complex or NUCB1 in coating buffer (0.2 M carbonate buffer, pH 9.5). After rinsing five times with ELISA wash buffer (0.1 M sodium phosphate, pH 7.5, 0.15 M NaCl, 0.05% Tween 20) the wells were treated for 2 hours with Protein Free Blocking Buffer and then incubated overnight with 50 µl of supernatant diluted in blocking buffer. After five washes, the wells were incubated for 1 hour with the detection antibody prepared in blocking buffer, washed and incubated for 1 hour with 50 µl HRP conjugated reaction antibody. After five washes, 50 µl of freshly made Amplex UltraRed (Invitrogen) substrate solution (5 µmol/L Amplex UltraRed in 50 mmol/L sodium citrate, pH 6.0 with the addition of H_2_O_2_) was added and HRP activity was detected by measuring fluorescence with a microplate reader.

#### 2.4.2 Conformation‐sensitive screening

To test the conformation‐sensitive property of the mAbs, their binding to Aβ40 monomers and Aβ42 protofibrils was measured through sandwich ELISA where mAbs were coated on Maxisorp 96‐well plates (NUNC) overnight at 4°C. After rinsing five times with ELISA wash buffer (0.1 M sodium phosphate, pH 7.5, 0.15 M NaCl, 0.05% Tween 20) the wells were treated for 2 hours with Protein Free Blocking Buffer. Antigen was added in blocking solution and the plates were treated as above.

### 2.5 Antibody production and purification

The hybridoma lines were cultured in Hi‐Growth Hybridoma Media (RPMI 1640 supplemented with 10% FBS, 10% NCTC‐109, 20 mmol/L HEPES, 2 mmol/L L‐glutamine, 1X NEAA, 50 IU/ml penicillin, 50 µg/ml streptomycin, and 1X Hybridoma Fusion and Cloning Supplement (Roche)). Supernatants were collected and antibodies were purified using a HiTrap Protein G HP column (GE Healthcare) with a running buffer of 20 mmol/L sodium phosphate, pH 7.0, and eluted with 100 mmol/L glycine‐HCl, pH 2.7 into faction tubes containing 1 M Tris, pH 9.0. Antibody containing fractions were pooled, dialysed with 1x DPBS (Life Technologies), concentrated with 30 kDa MWCO filters and stored in 1x DPBS, 10% glycerol, 0.02% NaN3. All mAbs were purified by SEC to remove protein aggregates and immediately used for the assays, in order to avoid potential artefacts due to avidity effect.

### 2.6 Fluorescence correlation spectroscopy

Fluorescence correlation spectroscopy (FCS) was performed using the ConfoCor 2 system (Carl Zeiss, Jena, Germany) consisting of an inverted microscope (Axiovert 200 M) equipped with a C‐Apochromat 40x NA = 1.2 water immersion UV‐VIS‐IR objective and avalanche photodiode detectors (SPCM‐AQR‐1X; PerkinElmer). Aβ40‐Alexa^488^ fluorescence was excited using the 488 nm line of the Argon ion laser. The main dichroic beam splitter HFT KP 700/488 was used to separate the incident and the emitted light. The pinhole size in front of the detector was 70 µm (1 Airy). The Aβ40‐Alexa^488^ peptide was diluted to a final concentration of 50 nmol/L in 20 mmol/L HEPES, pH 7.0. The 5C9.A2 binding was assessed through changes in the characteristic decay time of the temporal autocorrelation curve for Aβ40‐Alexa^488^. The anti‐Aβ 6E10^1^, ^2^, ^3^, ^4^, ^5^, ^6^, ^7^, ^8^, ^9^, ^10^, ^11^, ^12^, ^13^, ^14^, ^15^, ^16^ (Bio‐Legend, #803001) and IgG 1D4 (National Cell Culture Center) antibodies were used as positive and negative control, respectively. FCS measurements were performed at 20°C in multiple series. For each individual series, fluorescence intensity fluctuations were recorded in 10 consecutive measurements, each measurement lasting 10 seconds, and an average temporal autocorrelation curve was calculated.

Temporal autocorrelation curves were fitted using the theoretically derived autocorrelation function for a system that consists of a single molecular species with one triplet state when the molecules are undergoing free three‐dimensional (3D) diffusion:

$$G\left(\tau \right)=1+\frac{1}{N}\cdot \left[1+\frac{T}{1-T}\exp\left(-\frac{\tau }{\tau_{T}}\right)\right]\cdot \frac{1}{\left(1+\frac{\tau }{\tau_{D}}\right)\sqrt{1+\frac{w_{\mathrm{xy}}^{2}}{w_{z}^{2}}\frac{\tau }{\tau_{D}}}}$$

here, *N* is the average number of molecules in the observation volume element (OVE); *T* is the average equilibrium fraction of molecules in the triplet state; τ_T_ is the triplet correlation time; τ_D_ is the translational diffusion time; and *w_xy_* and *w_z_* describe the spatial distribution of the emitted light approximated by a three‐dimensional Gaussian, which is decayed to 1/e^2^ of its maximum intensity at *w_xy_* in the lateral direction and at *w_z_* in the axial direction. Calibration experiments using standard solutions of Rhodamine 6G (Rh6G) yielded τ_D, Rh6G_ = 26 ± 2 μs and (*w_xy_*/*w_z_*)^2^ = 7.^28^

To allow direct graphical comparison of characteristic decay times, the temporal autocorrelation curves were normalized to the same amplitude, G_n_(τ) = 1 at  = 10 µs.

### 2.7 Surface plasmon resonance

Surface plasmon resonance (SPR) studies were carried out with the ProteOn XPR36 protein interaction array system (Bio‐Rad) based on SPR technology.

The antibodies were immobilized in the vertical direction on GLM sensor chips (Bio‐Rad) using amine‐coupling chemistry, as described previously,^29^ followed by a blocking step with ethanolamine. The final immobilization level was ~6900 resonance units (1 resonance unit = 1 pg protein/mm^2^) for all the antibodies.

Successively, Aβ42 protofibrils obtained by incubating 10 µmol/L Aβ42 for 60 minutes at 37°C (diluted to 1, 0.5, 0.25, 0.125, 0.6 or 0 µmol/L), or freshly solubilized Aβ40 monomers (1, 0.3, 0.1 or 0 µmol/L) were flowed over the chip surface, in the horizontal direction, for 60 seconds at a flow rate of 30 µl/ml. The assays were performed at 25°C and the data were normalized by interspot and by buffer.

### 2.8 Thioflavin‐T binding assay

Nucleobindin 1 (NUCB1) inhibition of hIAPP aggregation was tested by using the Thioflavin‐T (ThT) fluorescence assay. Aggregation was monitored in presence of equimolar concentration of NUCB1 or the control protein BSA, and 10 µmol/L ThT (Fisher Scientific), for 24 hours at 25°C.

Similarly, the ability of the mAbs to inhibit hIAPP and Aβ42 aggregation was investigated by monitoring the kinetics of hIAPP and Aβ42 aggregation with or without mAbs. hIAPP and Aβ42 were diluted to 10 µmol/L concentration and 10 µmol/L ThT, with different concentrations of the antibodies and aggregation was monitored for 24 hours under quiescent conditions and constant temperature of 25 or 37°C, respectively*.*

In all cases, a volume of 50 µl per well (n = 4/group) was added to each well of a pre‐chilled (4°C) Corning 96 well half area black with clear flat bottom polystyrene with non‐binding surface (NBS) and covered with clear self‐adhesive topseal. Fluorescence measurements were performed on a Flexstation II (Molecular Devices) using an excitation wavelength of 450 nm and an emission wavelength of 485 nm. The obtained fluorescence measures were normalized to the relative fluorescence expressed after 30 minutes of incubation.

### 2.9 Protofibril staining in mouse cortex

15‐ to 16‐month‐old APP_102_/TTA mice (JAX 007004 and JAX 007051) were transcardially perfused and fixed with 4% paraformaldehyde (PFA). Whole brains were extracted and immediately placed in a 4% PFA solution at 4°C. The next day, brains were transferred to a 30% sucrose solution and stored at 4°C. Brains were frozen in optimum cutting temperature (OCT) blocks and sectioned at 10 µm. Slides were stored at −80°C until staining.

For immunohistochemistry, slides or the mouse prefrontal cortex were warmed to RT, then washed three times with 0.2% Triton‐X in PBS (PT) for 3 minutes each. Alternatively, when tissue was stained with LAMP2 (DSHB), a PBS‐saponin wash was used. Blocking was done in 5% goat serum for 1 hour at RT. The mAbs (4A8.E11, 4B1.H9, 3F2.E10, and 5C9.A2) were centrifuged at 15 000 x g for 5 minutes at 4°C before being diluted to a concentration of 10 µg/mL. Slides were co‐stained with rabbit polyclonal anti‐Aβ (1 µg/mL, Abcam #ab2539) antibody and were incubated overnight at 4°C. After incubation, slides were washed 3 times with PT for 20 minutes, then incubated for 2 hours at RT with the fluorescently‐conjugated secondary antibodies Alexafluor goat anti‐rabbit 488, Alexafluor goat anti‐mouse 594, Alexafluor goat anti‐rabbit 546, Alexafluor goat anti‐mouse 488 (Fisher). Slides were washed again and incubated in DAPI (1 µg/mL, Sigma) for 10 minutes. After a single 3‐minutes wash, slides were mounted and images were visualized on an inverted fluorescent microscope (AxioObserver Z.1; Zeiss, Thornwood, NY, USA). Animal tissue samples collection and the protocol used here were approved by the Baylor College of Medicine Institutional Animal Care and Use Committee (AN‐7033) and performed in accordance with regulations and established guidelines of the Stem Cells and Regenerative Medicine Center at Baylor College of Medicine.

### 2.10 Protofibril staining in human frontal cortex

All brain materials were obtained from the Huddinge Brain Bank at Karolinska Institutet Alzheimer Disease Research Center. All familial AD subjects met the criteria for definitive AD according to the Consortium to Establish a Registry for AD (CERAD).^30^ Serial 10 µm thick sections were exposed to antigen retrieval with DIVA Decloaker (DAKO) at 110°C for 30 minutes in a Decloaking Chamber NXGEN (BioCare Medical). Slides were then treated with peroxidase block (Dako) for 5 minutes at RT to quench endogenous peroxidase activity, and successively blocked with normal goat serum. The primary anti‐Aβ antibody (kindly provided by Jan Naslund^31^) or our mAbs were diluted in antibody diluent (Dako), followed by the EnVision mouse or rabbit antibody (Dako). 3,3’‐Diaminobenzidine (DAB) (Dako) was used to develop the slides for visualization using light microscopy. Human samples collection and the protocols used in the study were approved by the Stockholm ethical review board, unit 1 (Stockholms regional etikprövningsnämnd avdelning 1) with the reference number 2011/962‐13/1 on July 20, 2011 and all methods were performed in accordance with the relevant guidelines and regulation thereby established. The tissue was collected post‐mortem at the Brain Bank at Karolinska Institute upon voluntary donation and informed consent (informed consent forms are available upon request).

## 3 RESULTS

### 3.1 Immunization campaign and hybridoma screen for the discovery of tractable mAbs

**Figure 1:**
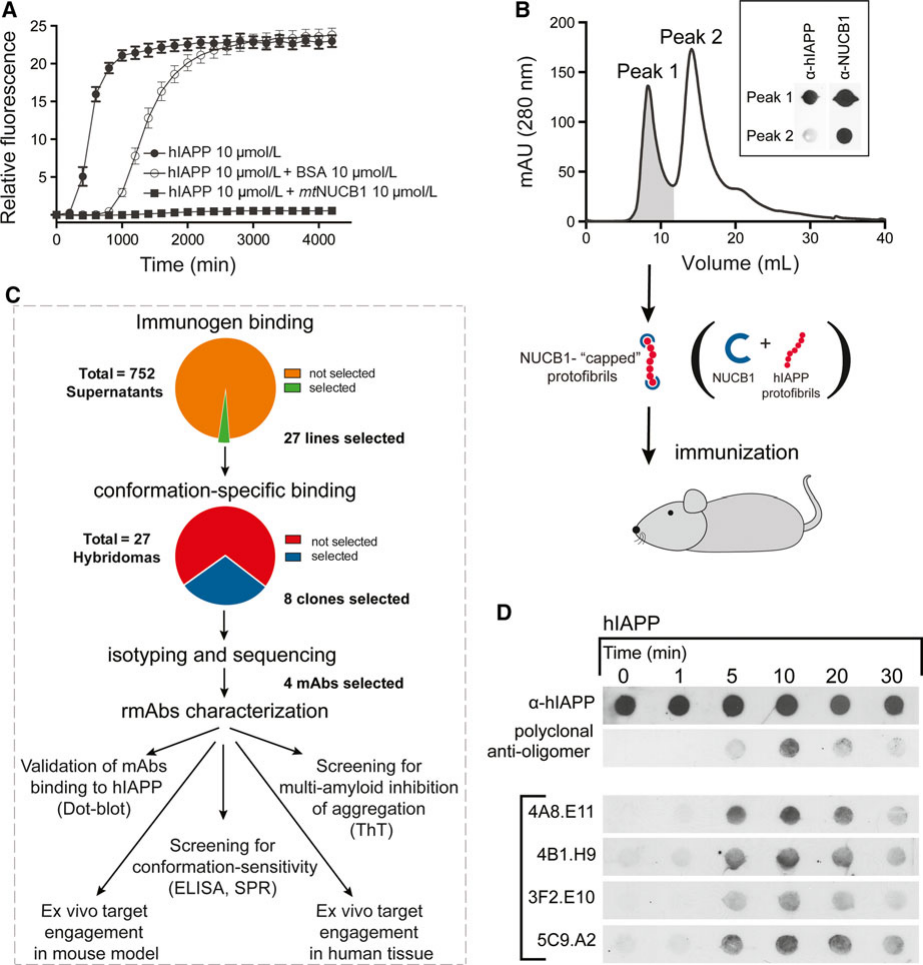
Production and validation of anti‐hIAPP monoclonal antibodies. A, The NUCB1‐mediated inhibition of hIAPP aggregation was monitored by the ThT assay. Time‐course graph shows the aggregation of hIAPP (10 µmol/L) alone and in the presence of equimolar concentration of BSA or NUCB1, incubated with 10 µmol/L ThT at 25°C. B, Size exclusion chromatography elution curve identifies two fractions of NUCB1‐hIAPP complexes of different size obtained by a mixture of hIAPP (33 µmol/L) and *mt*NUCB1 (10 µmol/L) incubated at 37°C for 3 h while stirred. The inset shows the reactivity of Peak 1 and Peak 2 to α‐hIAPP and α‐NUCB1 antibodies tested by dot blot. C, Schematic of the immunization strategy showing that Peak 1 composed of NUCB1‐capped hIAPP protofibrils was used to immunize mice. Out of 752 cell lines, 27 were selected based on their positive binding to NUCB1‐hIAPP complex and negative binding to NUCB1. Eight of these were isotyped and sequenced and 4 lines were selected for antibody purification and further studies. D, The monoclonal IgGs obtained from the selected hybridomas were validated by testing their binding to hIAPP by dot blot. The four mAbs (4A8.E11, 4B1.H9, 3F2.E10 and 5C9.A2), as well as the α‐hIAPP and the polyclonal anti‐oligomer A11 antibodies were tested on hIAPP (10 µmol/L) incubated for different time points (0, 1, 5, 10, 20 or 30 min)

The engineered Ca^2+^‐free NUCB1 inhibits hIAPP aggregation (Figure 1A) by binding to and stabilizing short protofibrils (Figure S1), as previously reported.^27^ The hIAPP‐NUCB1 complex was purified using SEC (Figure 1B), characterized by atomic force microscopy (AFM) and ELISA (Figure S1) and injected into three mice that showed robust titre to the immunogen (Figure S2A). The immunization campaign (Figure 1C) resulted in 752 screened fusion wells that were subjected to a pre‐subclone screen by ELISA for their reactivity to the immunogen complex NUCB1‐hIAPP as well as to NUCB1 (Figure S2B). Based on their binding profile we selected 27 lines for expansion and single‐point analysis for reactivity to Aβ42 protofibrils (results not shown).

We chose and sub‐cloned eight lines (5G10.A2, 5A8.B11, 1G3.B12, 7B6.B12, 4A8.E11, 4B1.H9, 3F2.E10 and 5C9.A2) that showed desired characteristics. Isotyping and sequencing results showed that 7B6.B12 was an IgM, and that 5A8.B11 and 1G3.B12 were mixed clones (not shown). 5G10.A2 showed the same sequence as 4B1.H9 (not shown) and was therefore discarded. The remaining four monoclonal IgGs (4A8.E11, 4B1.H9, 3F2.E10 and 5C9.A2) were selected for antibody purification.

### 3.2 Validation of mAbs binding to hIAPP intermediate aggregates

We first tested the purified mAbs for binding to hIAPP protofibrils by dot blot. The results indicate that all mAbs bind to hIAPP, preferentially to the protofibril species that form after 5‐20 minutes of incubation, similarly to the binding signal shown by the polyclonal anti‐oligomer antibody A11 (Invitrogen # AHB0052), used as control (Figure 1D).

### 3.3 Screening of conformation‐sensitive, sequence‐independent mAbs

**Figure 2:**
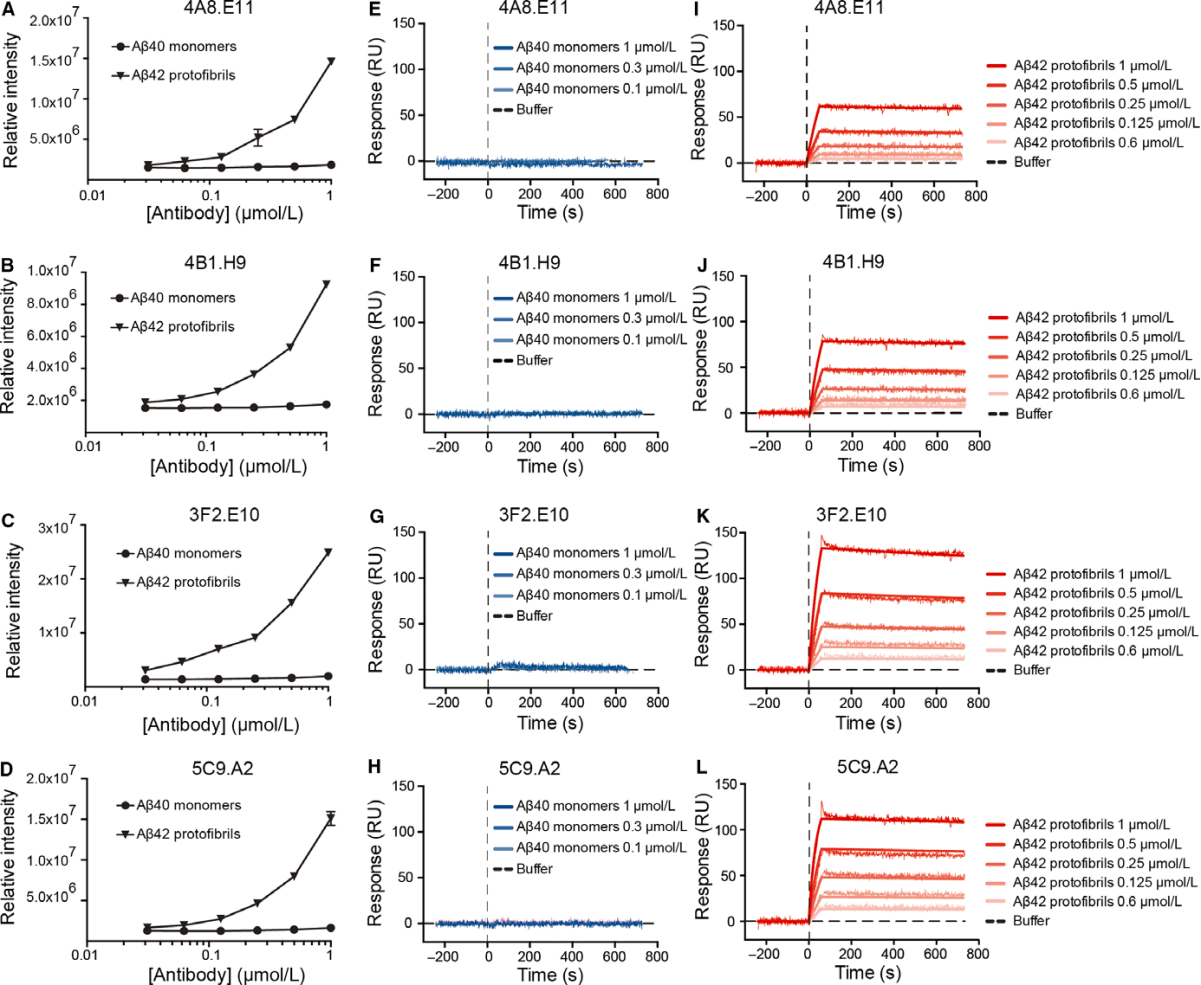
Conformation‐sensitive mAbs bind Aβ42 protofibrils but not unstructured Aβ40 monomers. A‐D) A sandwich enzyme‐linked immunosorbent assay (ELISA) was used to test the binding of A) 4A8.E11, B) 4B1.H9, C) 3F2.E10 and D) 5C9.A2 mAbs to Aβ42 protofibrils or Aβ40 monomers. The antibodies were coated on the plate and used to capture either freshly prepared Aβ40 (monomers) or Aβ42 that had been incubated at 37°C for 30 min to enrich protofibril species. Data are normalized to the lowest antibody concentration. E‐L) The SPR was also used to test the mAbs binding to E‐H) Aβ40 monomers and I‐L) Aβ42 protofibrils. Freshly solubilized Aβ40 monomers (10 µmol/L) and Aβ42 protofibrils (10 µmol/L) were flown at different concentrations for 60 s over each antibody (300 nmol/L) previously immobilized on the chip (RL = 6500). Data are normalized by interspot and buffer and presented as mean ± standard error of the mean (SEM)

To test our hypothesis that NUCB1‐hIAPP originated mAbs would detect the common quaternary amyloid protofibril structure independently from the primary sequence, we analysed whether they cross‐react with structured protofibrils originating from the Aβ42 peptide. Sandwich ELISA and FCS were used to determine whether mAbs bind Aβ monomers or protofibrils. Sandwich ELISA shows that each mAb specifically binds to the protofibril enriched pools of Aβ42 antigen and not to Aβ40 monomers (Figure 2A‐D, left column). These data suggest that the conformation‐sensitive anti‐hIAPP mAbs specifically recognize the protofibril conformation in a sequence‐independent way.

FCS, performed for 5C9.A2 only, confirmed the results obtained by sandwich ELISA, showing that the mAb 5C9.A2 does not bind to Aβ40‐Alexa^488^ monomers even when the mAb is present in a large excess (100:1), as evident from the unchanged characteristic decay time of the temporal autocorrelation curves that agrees well with the diffusion time of Aβ40‐Alexa488 alone [τ_D_, Aβ40‐Alexa488 = 67 ± 3 µs, obtained across all experiments, n = 110] (Figure S3A). In contrast, the anti‐Aβ 6E10^1^, ^16^ mAb used as positive control, readily binds to Aβ40‐Alexa^488^ monomers, even at low antibody/antigen ratio (1:10), as evident from the rightward shift of the temporal autocorrelation curve to longer lag time values [_D, Aβ40‐Alexa488 /6E10_ = 212 ± 70 µs], indicating an increase in diffusion time when Aβ40‐Alexa^488^ is bound to the mAb (Figure S3B). As expected, the IgG 1D4 mAb, used as negative control, did not bind Aβ_40_‐Alexa^488^, as indicated by the unaltered decay time of the temporal autocorrelation curves compared to the diffusion time of Aβ40‐Alexa^488^ alone (Figure S3C).

Furthermore, in order to avoid possible artefacts due to fluorescence labeling and to more precisely characterize mAbs binding, the label free SPR assay was employed. A direct binding experiment was performed by immobilizing the mAbs on the chip surface and flowing different dilutions of Aβ42 protofibrils (1, 0.5, 0.25, 0.125, 0.06 or 0 µmol/L) or Aβ40 monomers (1, 0.3, 0.1 or 0 µmol/L).

We observed a strong, concentration‐dependent binding to Aβ42 protofibrils for all mAbs (Figure 2I‐L, right column), as well as for the positive control, anti‐Aβ antibody 6E10 (Figure S4A,C), but not the negative control 1D4 (Figure S4B,D). Notably, the shape of the dissociation curve indicates a strong association of the binding clones to the protofibrils that does not spontaneously resolve during the washing phase with NaCl (740 seconds). Furthermore, we show that the anti‐Aβ antibody 6E10 (Figure S4A, K_d_ = 4.66 nmol/L), but none of our mAbs (Figure 2E‐H, centre column) or the negative control 1D4 (Figure S4B), concentration‐dependently binds Aβ40 monomers.

The SPR assay provides accurate, label‐free measurement of the binding kinetics. However, in the case of antibody‐amyloid binding, the data analysis requires particular care. In fact, the correct immobilization of the antibody on the chip surface is a critical step to guarantee good quality data. The ideal immobilization level (R_L_) is calculated according to the equation:

$$R_{L}=\frac{M_{W_{Ligand}}}{M_{W_{Analyte}}}\cdot R_{max}\cdot \frac{1}{n}$$

where *Ligand* is the antibody and *Analyte* is the amyloid (protofibril or monomer), *Mw* is the molecular weight, *R_max_* indicates the maximum theoretical response of the analyte (200) and n represents the stoichiometry of the reaction. When the analyte is the amyloid monomeric sample, the equation can be easily solved, due to the known Aβ42 monomer molecular weight (4.5 kDa) and known stoichiometry (n = 1). On the contrary, when amyloid protofibrils are the analyte, both the protofibril molecular weight and the stoichiometry of binding are unknown and therefore the ideal immobilization level cannot be calculated. Notably, a sub‐optimal immobilization of the antibody to the chip surface does not make the assay prone to false positive or false negative results, but rather makes the binding kinetics results hard to assess.

In summary, binding of 4A8.E11, 4B1.H9, 3F2.E10, and 5C9.A2 mAbs to Aβ42 protofibrils, but not to Aβ40 monomers, suggests selectivity of these mAbs to the protofibril state of the amyloid, indicating sequence‐independent, conformation‐selective binding (Figure 2E‐L).

### 3.4 Conformation‐sensitive mAbs inhibit hIAPP and Aβ42 aggregation

In order to determine if the antibody binding to amyloid aggregates has a functional effect on fibrillization, a ThT assay was used to measure aggregation kinetics. The assay is based on the alteration that the ThT fluorescence spectrum encounters upon binding to amyloid and therefore the fluorescent signal is considered a measure of protein aggregation.

**Figure 3:**
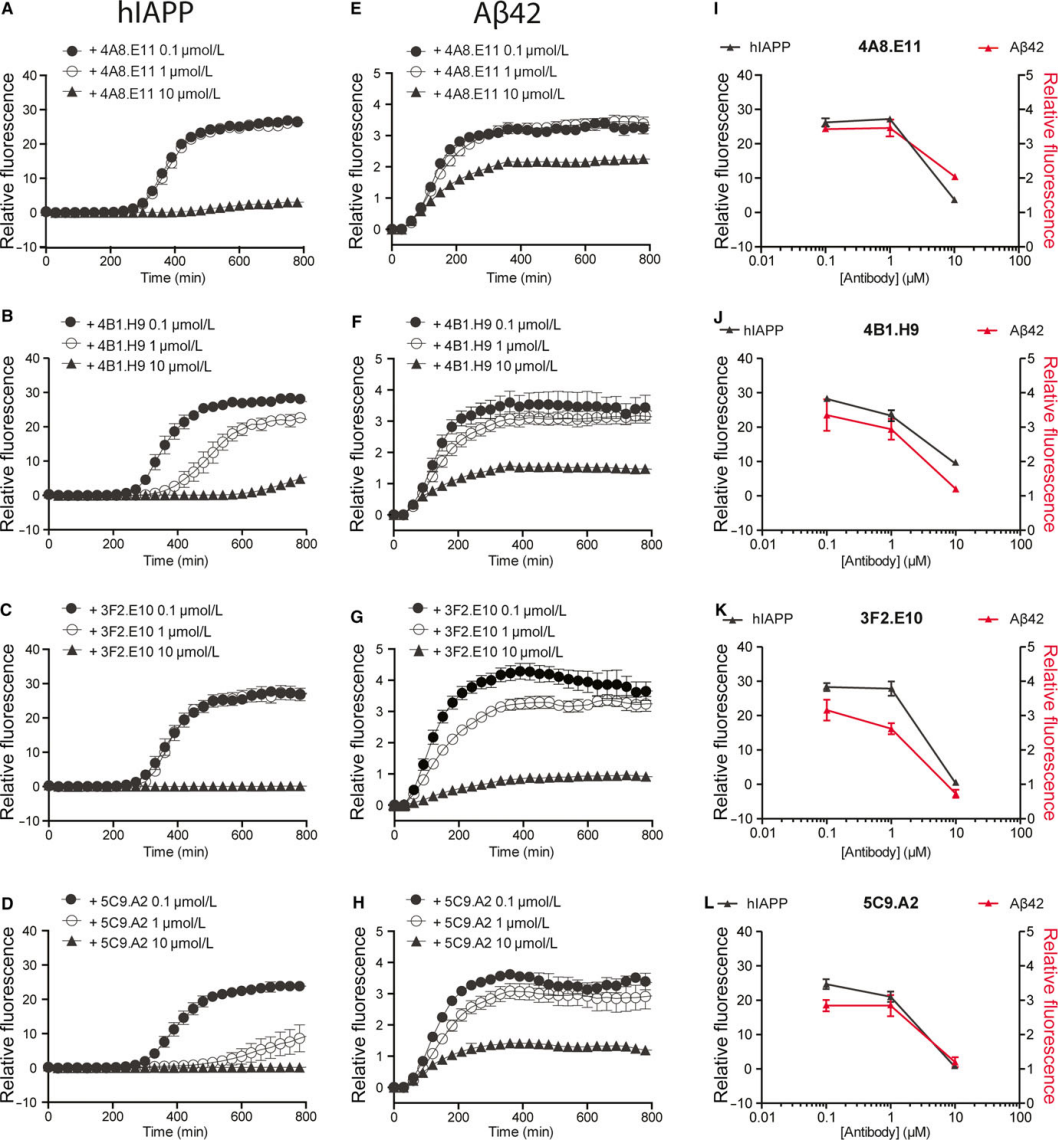
mAbs inhibit hIAPP and Aβ42 aggregation. The mAbs‐mediated inhibition of hIAPP and Aβ42 aggregation was monitored by the ThT assay. Time course graphs show the aggregation of A‐D) hIAPP (10 µmol/L) and E‐H) Aβ42 (10 µmol/L) incubated with 10 µmol/L ThT at 25 and 37°C, respectively, alone or together with different concentrations of A, E) 4A8.E11; B, F) 4B1.H9; C, G) 3F2.E10 and D, H) 5C9.A2. I‐L) Data shown in A‐H are plotted together for direct comparison of the inhibition exerted by I) 4A8.E11; J) 4B1.H9; K) 3F2.E10 and L) 5C9.A2 on the aggregation of hIAPP (10 µmol/L, black) and Aβ42 (10 µmol/L, red). Data are expressed as relative fluorescence and presented as mean ± SEM

The results indicate a concentration‐dependent inhibitory effect of the 4A8.E11, 4B1.H9, 3F2.E10, and 5C9.A2 antibodies on the aggregation of hIAPP (Figure 3A‐D) or Aβ42 monomers (Figure 3E‐H), compared with the negative control IgG 1D4 antibody (Figure S5). Notably, the minimal decrease in fluorescence exerted by the negative control is a commonly reported effect of the protein mass, also observed when hIAPP aggregates in the presence of BSA (Figure 1). When the data were plotted together, it appeared that the antibodies have a comparable inhibitory effect on the two amyloid proteins (Figure 3I‐L), except for 3F2.E10 that acts more potently on Aβ42 aggregation (Figure 3K). We hypothesized that the sequence‐independent inhibition of amyloid aggregation may occur through functional binding of the antibodies to early pre‐fibrillar aggregates, preventing them from maturing to the fibril state.

### 3.5 Protofibril staining in APP_102_/TTA brain tissue

**Figure 4:**
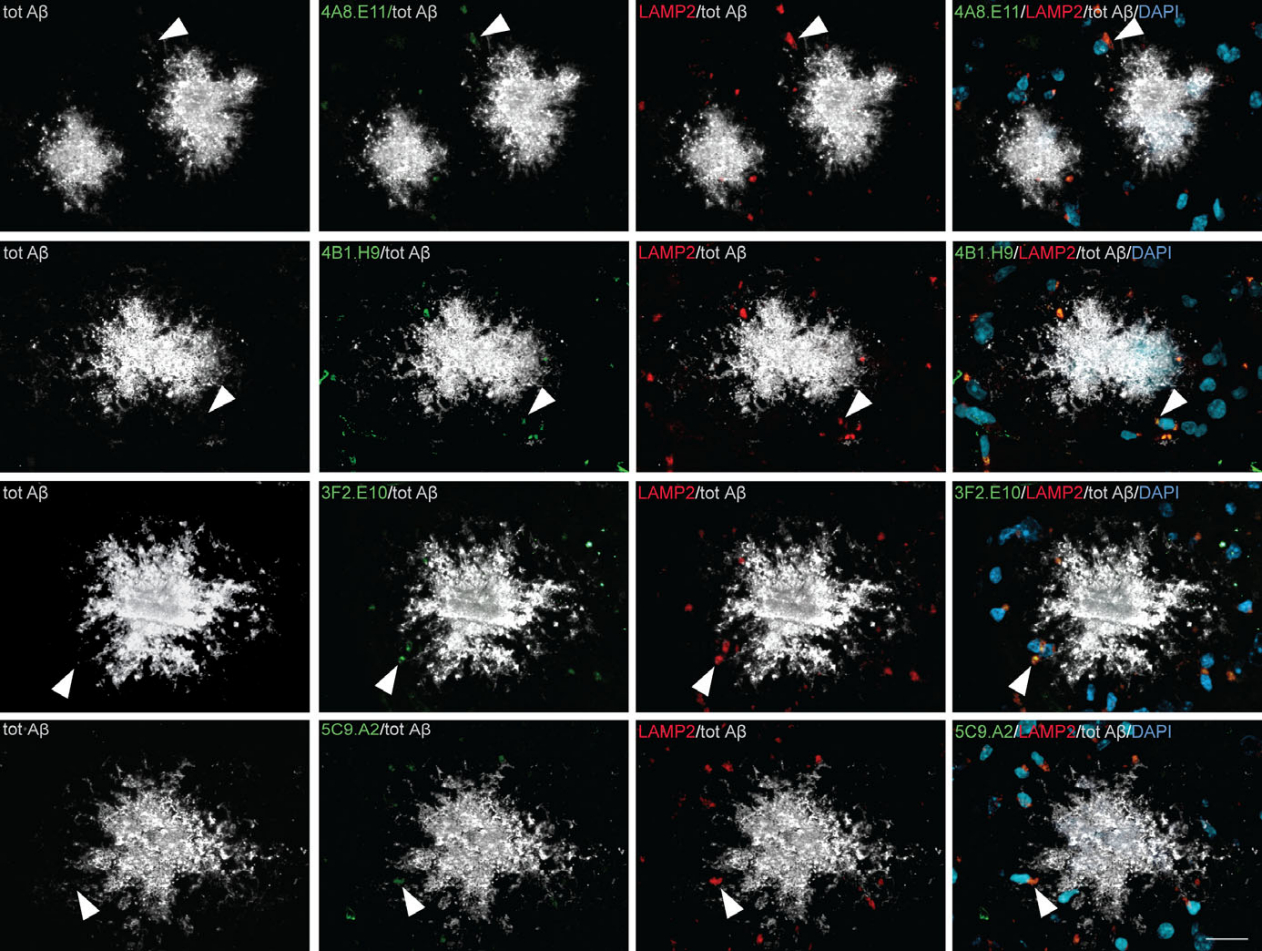
mAbs reveal punctate staining around plaques but don't associate with the plaques in an AD mouse model cortex. The brain cortex obtained from APP102/TTA mice was stained to analyze the localization of our mAbs‐binding signal. Immunohistochemistry reveals that mAbs 4A8.E11, 4B1.H9, 3F2.E10, and 5C9.A2 (green) detect small, subdiffraction‐limited spot size regions surrounding the total α‐Aβ antibody‐positive plaques (grey). mAbs signal co‐localizes with the lysosome marker LAMP2 (red) localized in the vicinity of cell nuclei stained by the neuronal marker DAPI (dark cyan), but not with the plaques. Arrowheads indicate spots where co‐localization was observed; scale bar = 20 µm

To test the ability of these mAbs to detect protofibrils in tissue, we tested target engagement in ex vivo tissue from a transgenic mouse model of AD. The cortex of APP_102_/TTA mice was sectioned and co‐stained with polyclonal anti‐Aβ antibody, our mAbs (4A8.E11, 4B1.H9, 3F2.E10, and 5C9.A2), as well as the lysosome membrane marker LAMP2 and the nuclear marker DAPI (Figure 4). We observed that the mAbs staining was present in proximity, but not inside of the Aβ plaques stained by the total anti‐Aβ antibody in the transgenic mice (Figure 4), but not in wild‐type (WT) tissue or regions with no plaques (not shown). Furthermore, we observed co‐localization of the mAbs staining with LAMP2 as well as DAPI (Figure 4). This staining pattern suggests that our mAbs do not bind to the Aβ species that deposit in plaques but only to intracellular species localized in the lysosome.

### 3.6 Intraneuronal staining in human AD frontal cortex

**Figure 5:**
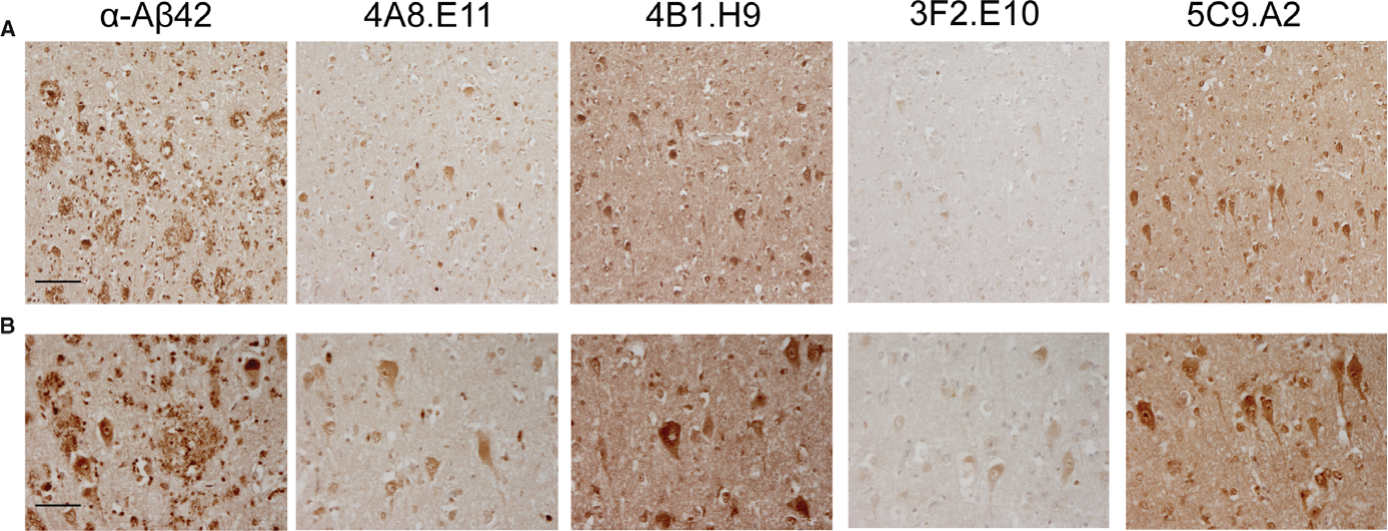
Conformation‐specific mAbs stain pyramidal cells in AD human frontal cortex. The brain cortex obtained from familial AD patients was stained to analyze the localization of our mAbs‐binding signal. DAB immunohistochemistry reveals that while the α‐Aβ42 antibody stains plaques, 4A8.E11, 4B1.H9, 3F2.E10, and 5C9.A2 do not bind to plaques but specifically stain pyramidal cells. Scale bar = 500 µm in A and 250 µm in B

Frontal cortex brain tissue obtained from a familial AD patient was serially sectioned and adjacent slices were stained with the positive control anti‐Aβ42 antibody or our conformation‐sensitive mAbs to test the signal localization using immunohistochemistry. We observed that while the anti‐Aβ42 antibody stained both plaques and pyramidal neurons in the AD frontal cortex, none of our mAbs detected plaques, but specifically and strongly stained pyramidal cells (Figure 5). These results suggest that the pyramidal neurons contain Aβ protofibril morphotypes, whereas the Aβ‐dense plaques do not contain these structures, therefore strengthening the results obtained in animal tissue and confirming the conformation‐sensitive feature of our mAbs.

## 4 DISCUSSION

In the past decades, the pathologic role of toxic protofibrils in the development of amyloid diseases has been increasingly recognized,^32^, ^33^ leading to the study of therapeutics targeting early amyloid species to prevent large aggregates and plaque formation. Many approaches have been used to develop conformation‐sensitive anti‐amyloid antibodies to reduce toxicity and prevent further aggregation. Currently, encouraging results arise from clinical trials with BAN2401 (BioArtic Neuroscience AB, Eisai Co., Ltd.), crenezumab (Genentech, Inc) and aducanumab (Biogen, Inc), in Phase II, III and III, respectively. Although these mAbs have been produced through different approaches, they all rely on the therapeutic hypothesis that soluble intermediate aggregates (protofibrils) are the most toxic species in the Aβ fibrillization pathway.^32^

Aside from the biologics currently in clinical trials, the polyclonal antibody A11, described more than 10 years ago, has been a useful tool for researchers for detecting the oligomeric species of amyloid. This polyclonal antibody was produced using the C‐terminal thioester Aβ40 monomers tethered to gold colloid nanoparticles as an immunogen.^18^ Alternatively, the monoclonal WO1 and WO2 antibodies were created using sonicated fibrils and react to the general structure of fibrils from many amyloid sources.^34^ The murine version of BAN2401, mAb158, was produced using protofibrils obtained from a mutated Aβ42 peptide (E22G, Arctic mutation).^35^ Clone 13C3 was created using Aβ42 fibrils followed by screening for protofibril reactivity.^36^ Recently, an anti‐Aβ42 polyclonal antibody has been obtained by immunizing animals with isolated Aβ42 protofibrils.^37^

In contrast to these approaches, we have created a platform technology using the novel NUCB1‐capping method to produce stable amyloid protofibrils^27^, ^34^ that can be used as immunogen to create panels of conformation‐sensitive mAbs. We hypothesized that our technology could be used to produce conformation‐sensitive, sequence‐independent mAbs that detect the quaternary structure of the protofibrils and bind to early amyloid aggregates independently from the primary structure.

In this work we show that immunizing animals with hIAPP protofibrils stabilized by the CLABP, NUCB1, can produce mAbs that bind both hIAPP and Aβ protofibrils and we describe the methodology used to screen for mAbs with this conformation‐sensitive feature. Binding assays, such as dot blot (Figure 1D), ELISA (Figure 2A‐D), SPR (Figure 2E‐L) and FCS (Figure S3), show that four mAbs bind to hIAPP and Aβ42 protofibrils but not monomers (Figures 2A‐D and S3).

Successively, we tested the functional binding of these mAbs to Aβ42 prefibrillar species by analyzing the kinetics of aggregation of the peptide with and without different concentrations of the mAbs (Figure 3). The ThT data indicate that these mAbs inhibit aggregation of hIAPP and Aβ42. We hypothesized that the inhibition of amyloid aggregation may occur through functional binding of the antibodies to early aggregates, preventing them from maturing to the fibril state, though further studies are needed to elucidate the antibodies mechanism of inhibition.

These novel anti‐protofibril mAbs have shown, in a disease‐specific animal model of AD, staining around, but not inside the Aβ plaques in the prefrontal cortex. This staining pattern appears to be intracellular and granular. There has been debate as to whether intracellular or extracellular amyloid aggregation is a major driver of plaque formation.^38^ When co‐stained with the lysosomal marker LAMP2 the mAbs display a co‐localization pattern suggesting that protofibrils accumulate within lysosomes. These studies indicate that our mAbs co‐stain intracellular lysosomal vesicles that contain Aβ and protofibril conformations, supporting the model of intracellular protofibril formation or accumulation.

We further studied these mAbs using human familial AD frontal cortex samples. The AD tissue displayed Aβ‐positive plaques and Aβ‐positive intraneuronal staining pattern. Using serial sections and imaging near the same area, we found that our mAbs do not display a plaque‐like pattern, but rather showed a specific intracellular pyramidal neuron staining. These data support the mouse tissue staining pattern and therefore we conclude that our mAbs do not react with Aβ‐positive plaque, but specifically bind to an intraneuronal protofibril conformation.

In summary, this work shows that our platform technology of NUCB1‐capped hIAPP protofibrils is a suitable tool for discovering mAbs with different reactivity profiles. We provide a detailed screening platform to assess functional binding and target engagement in in vitro and ex vivo models. These anti‐protofibril antibodies (a) detect specific structural elements from at least two different amyloid sources, (b) capture soluble protofibrils in solution, (c) disrupt normal aggregation kinetics, and (d) reveal valuable insight into to the localization of protofibrils in tissues of either models of disease or that of tissue from humans who were afflicted with disease.

## CONFLICT OF INTEREST

The authors declare that they have no competing interests.

## AVAILABILITY OF DATA AND MATERIALS

The datasets used and/or analysed during the current study are available from the corresponding author on reasonable request.

## BIOETHICAL STATEMENT

Animal tissue samples collection and the protocol used in this study were approved by the Baylor College of Medicine Institutional Animal Care and Use Committee (AN‐7033) and performed in accordance with regulations and established guidelines of the Stem Cells and Regenerative Medicine Center at Baylor College of Medicine. Human samples and their use in the study were approved by the Stockholm ethical review board, unit 1 (Stockholms regional etikprövningsnämnd avdelning 1) with the reference number 2011/962‐13/1 on July 20, 2011.

## AUTHOR CONTRIBUTIONS

ABO, WVG and TPS designed the study. ABO and WVG prepared and characterized the immunogen, supervised the immunization campaign, and conducted the in vitro experiments. SSW, CG and LOT performed the immunohistochemical studies on human tissue, and SSY and RP performed the studies on animal tissue. AT, VV and LT carried out the fluorescence correlation spectroscopy study. CA provided helpful input for the surface plasmon resonance experiments. NP, MB and YR performed the atomic force microscopy experiments. BW provided essential insights throughout the entire study. ABO and WVG wrote the paper with critical input from TPS and all other authors who approved the final version.
