# Supplementary figures and images for: Conformation‐specific antibodies against multiple amyloid protofibril species from a single amyloid immunogen
Source: J Cell Mol Med. 2019 Jan 20;23(3):2103–14. doi: 10.1111/jcmm.14119 (PMC6378190; doi:10.1111/jcmm.14119)

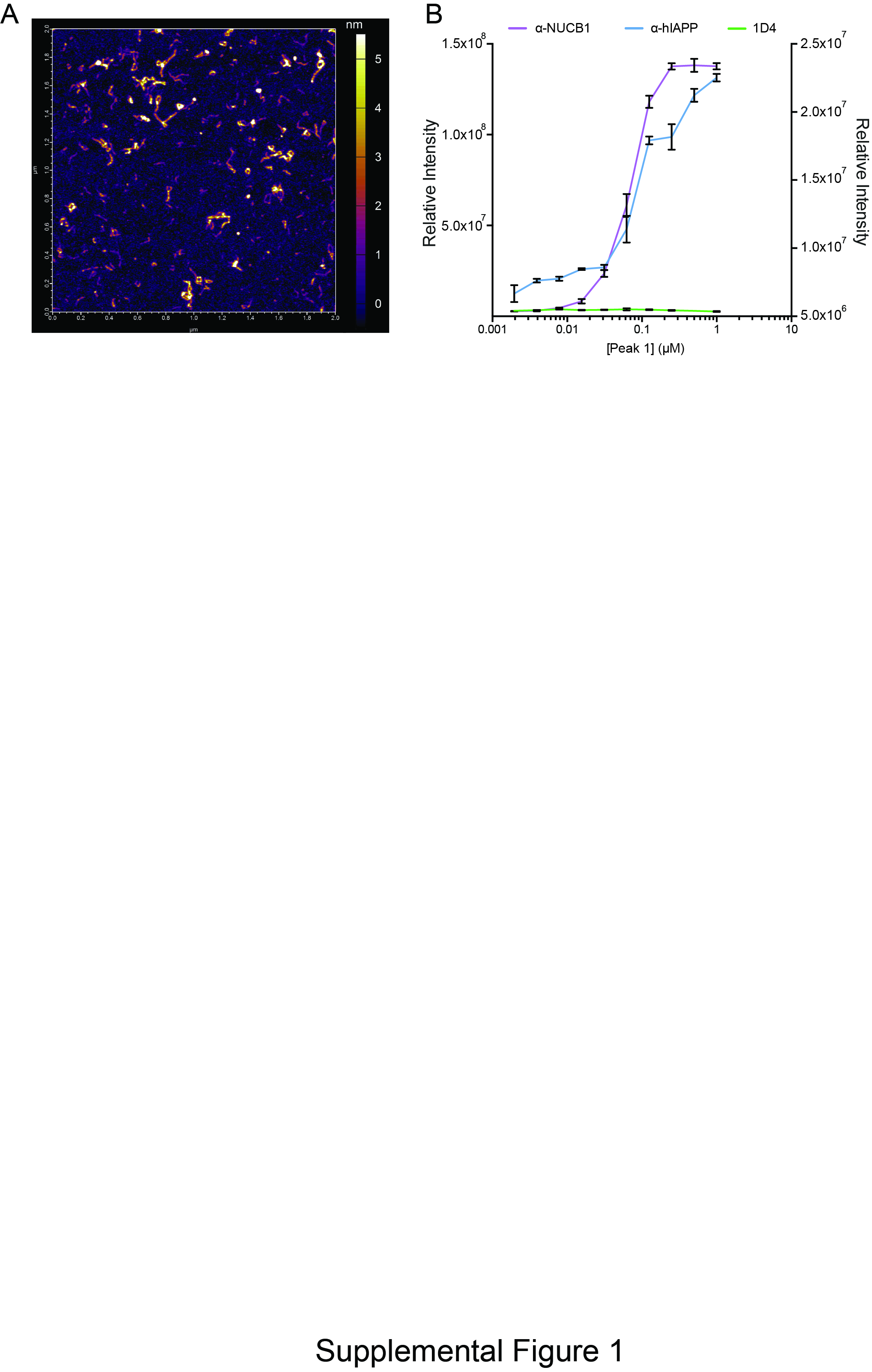

Supplement: Supplementary file 1 [file JCMM-23-2103-s001.tif]

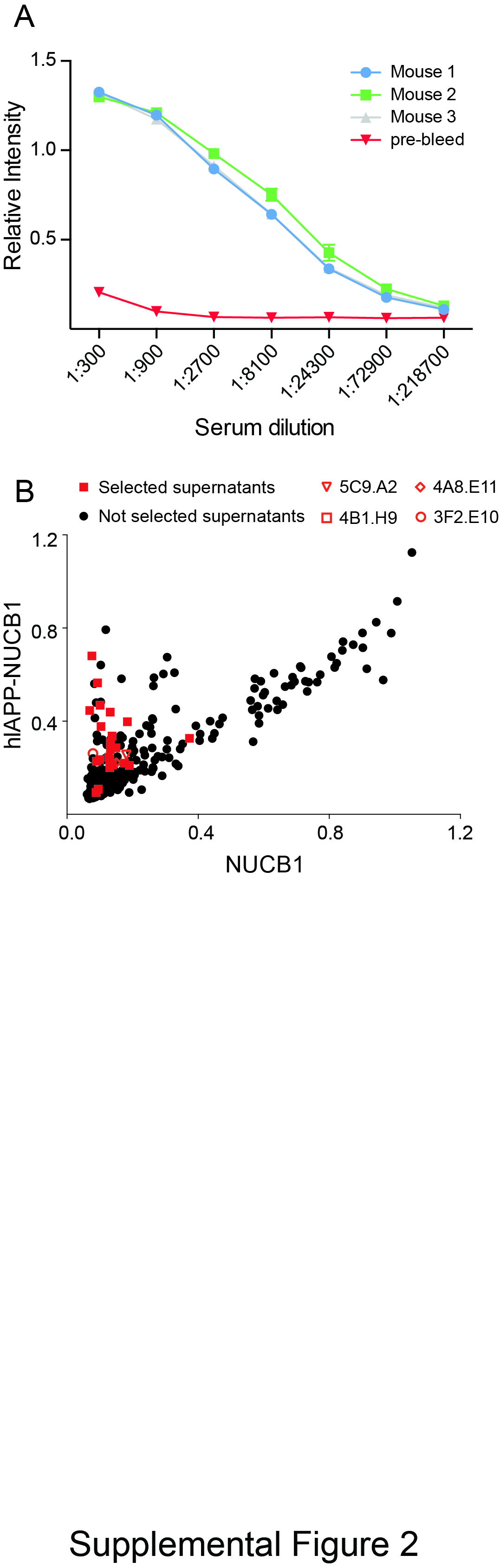

Supplement: Supplementary file 2 [file JCMM-23-2103-s002.tif]

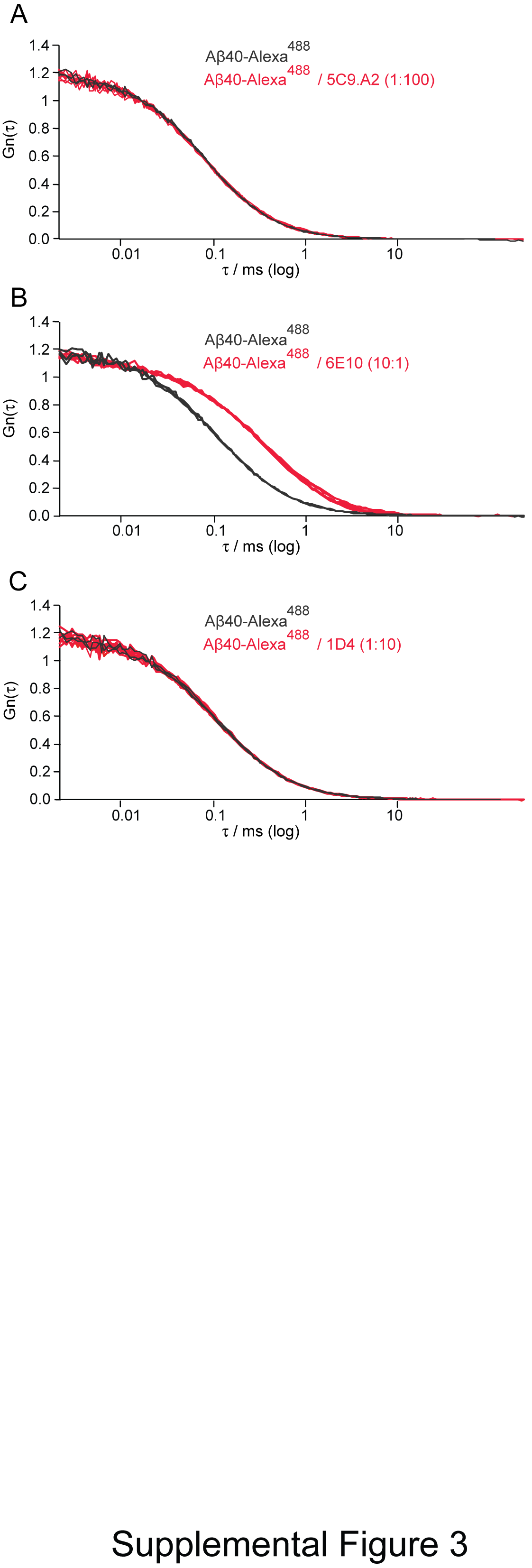

Supplement: Supplementary file 3 [file JCMM-23-2103-s003.tif]

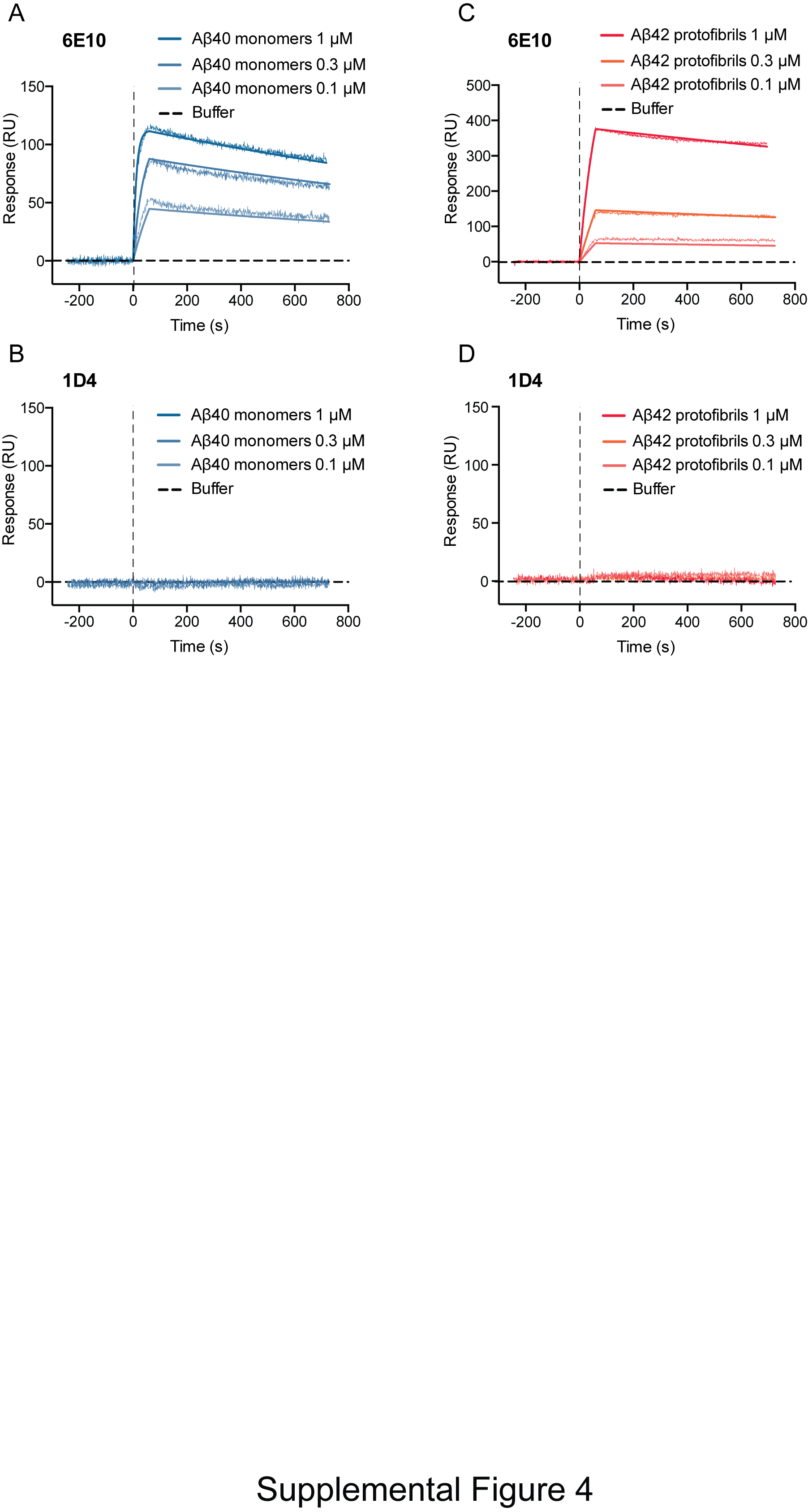

Supplement: Supplementary file 4 [file JCMM-23-2103-s004.tif]

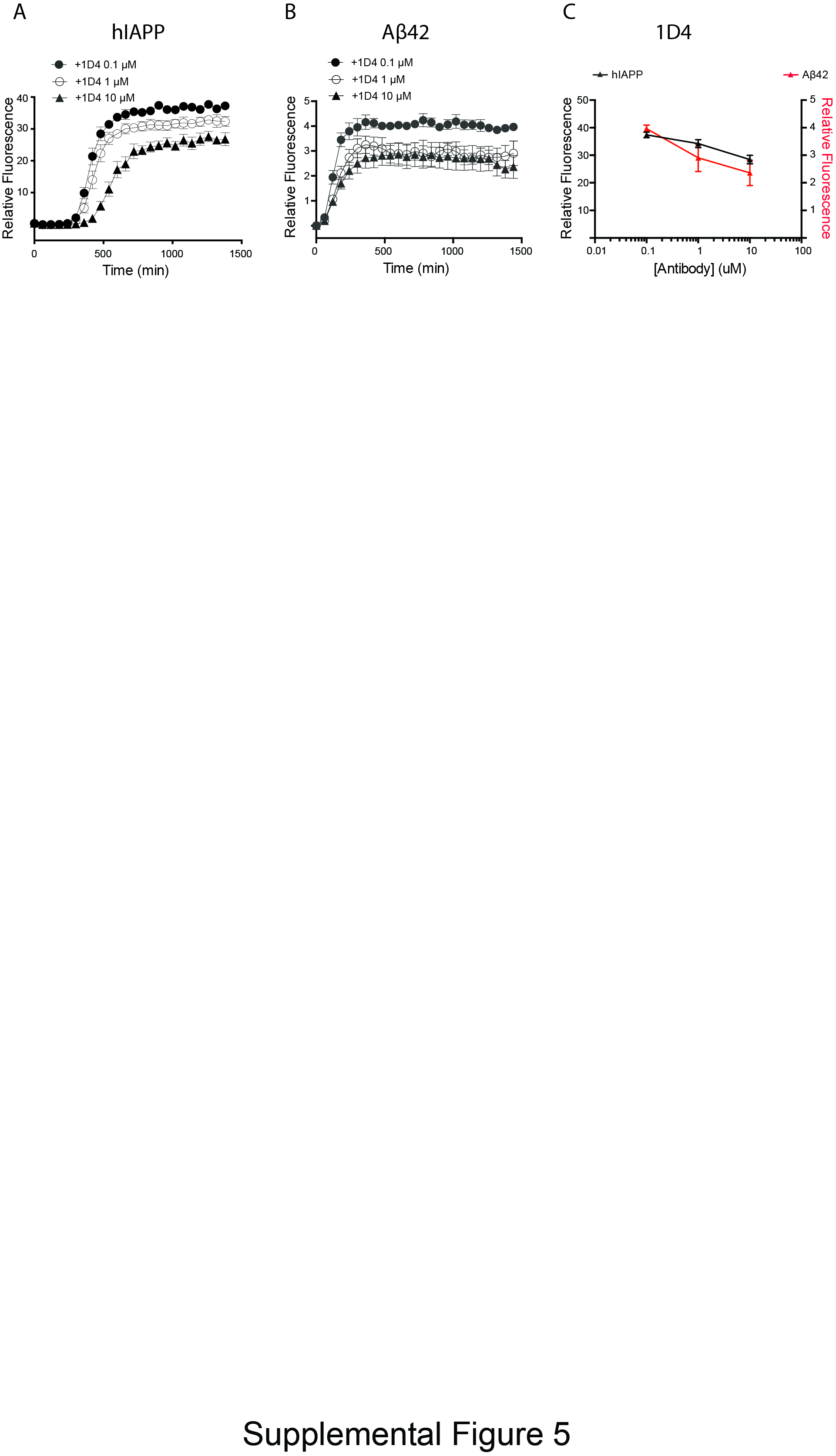

Supplement: Supplementary file 5 [file JCMM-23-2103-s005.tif]
