# Supplementary material for: Conformation‐specific antibodies against multiple amyloid protofibril species from a single amyloid immunogen
Source: J Cell Mol Med. 2019 Jan 20;23(3):2103–14. doi: 10.1111/jcmm.14119 (PMC6378190; doi:10.1111/jcmm.14119)
Supplement: Supplementary file 6 [file JCMM-23-2103-s006.docx]

**Conformation-specific Antibodies Against Multiple Amyloid Protofibril Species from a Single Amyloid Immunogen**

**Alessandra Bonito-Oliva^1^, Sophia Schedin-Weiss^2^, Shahab S. Younesi^3^, Ann Tiiman^4^, Carolina Adura^5^, Navid Paknejad^6^, Matt Brendel^6^, Yevgeniy Romin^6^, Ronald J. Parchem^3^, Caroline Graff^2^, Vladana Vukojević^4^, Lars O. Tjernberg^2^, Lars Terenius^4^, Bengt Winblad^2^, Thomas P. Sakmar^1, 2^, W Vallen Graham^1^**

**^1^**Laboratory of Chemical Biology & Signal Transduction, The Rockefeller University, New York, NY 10065

**^2^** Department of Neurobiology, Care Sciences and Society, Center for Alzheimer Research, Division of Neurogeriatrics, Karolinska Institutet, 14157 Huddinge, Sweden

**^3^** Department of Neuroscience, Stem Cells and Regenerative Medicine Center, Baylor College of Medicine, Houston, TX, US

**^4^** Department of Clinical Neuroscience, Center for Molecular Medicine, Karolinska Institutet, 17176 Stockholm, Sweden

^5^ High Throughput and Spectroscopy Resource Center, The Rockefeller University, 1230 York Avenue, New York 10065

^6^ Molecular Cytology Core Facility, Memorial Sloan-Kettering Cancer Center, New York, NY, USA

**Suppl. Fig. 1 Screening of NUCB1-hIAPP immunogen complex.** The NUCB1-hIAPP complex used to immunize the mice was characterized by **A)** AFM and revealed the presence of short protofibrils. The color bar indicates the height of the species. **B)** The direct ELISA assay shows that the NUCB1-hIAPP complex contained in the SEC Peak 1 shows positive binding to both the α-hIAPP and the α-NUCB1 antibodies. Data are expressed as mean ± SEM.

**Suppl. Fig. 2 Immunization results and clones screening. A)** The mouse titers after immunization were analyzed and compared with the pre-bleed values. **B)** 752 post-fusion supernatants were analyzed for reactivity to either NUCB1 alone or the NUCB1-hIAPP immunogen. 27 lines were selected for further analysis.

**Suppl. Fig. 3 FCS assay confirms that 5C9.A2 does not bind to the unstructured Aβ40 monomers.** Temporal autocorrelation curves recorded in a 50 nM Aβ40-Alexa^488^ solution alone or in presence of **A)** 5C9.A2 (5 µM), **B)** the positive control α-Aβ 6E10 [1,16] (5 nM) or **C)** the negative control 1D4 (500 nM). The antibody/antigen ratio is as indicated.

**Suppl. Fig. 4 Binding of the positive and negative control antibodies to Aβ protofibrils in the SPR assay. A, B)** freshly prepared Aβ40 monomers (10 µM) or **C, D)** Aβ42 protofibrils (10 µM) were flown at different concentrations for 60 sec over **A, C)** the positive control, α-Aβ 6E10 antibody (300 nM) or **B, D)** the negative control, irrelevant IgG 1D4 antibody (300 nM) previously immobilized on the chip (RL = 6500). Data are normalized by interspot and buffer and presented as mean ± SEM.

**Suppl. Fig. 5 Inhibitory effect of the negative control antibody on hIAPP and Aβ42 aggregation.** The inhibition of hIAPP and Aβ42 aggregation was monitored by the ThT assay. Time course graphs show the aggregation of **A)** hIAPP (10 µM) and **B)** Aβ42 (10 µM) incubated with 10 µM ThT at 25 ^o^C and 37 ^o^C, respectively, in the presence of different concentrations of the negative control antibody, 1D4. **C)** Data shown in A, B are plotted together for direct comparison of the inhibition exerted 1D4 on the aggregation of hIAPP (10 µM, black) and Aβ42 (10 µM, red). Data are expressed as relative fluorescence and presented as mean ± SEM.
